# COVID-19 outcomes in people living with HIV: Peering through the waves

**DOI:** 10.1016/j.clinsp.2023.100223

**Published:** 2023-05-25

**Authors:** Thaís Lorenna Souza Sales, Maíra Viana Rego Souza-Silva, Polianna Delfino-Pereira, João Victor Baroni Neves, Manuela Furtado Sacioto, Vivian Costa Morais de Assis, Helena Duani, Neimy Ramos de Oliveira, Natália da Cunha Severino Sampaio, Lucas Emanuel Ferreira Ramos, Alexandre Vargas Schwarzbold, Alzira de Oliveira Jorge, Ana Luiza Bahia Alves Scotton, Bruno Mateus de Castro, Carla Thais Cândida Alves da Silva, Carolina Marques Ramos, Fernando Anschau, Fernando Antonio Botoni, Genna Maira Santos Grizende, Guilherme Fagundes Nascimento, Karen Brasil Ruschel, Luanna Silva Monteiro Menezes, Luís César de Castro, Luiz Antônio Nasi, Marcelo Carneiro, Mariana Frizzo de Godoy, Matheus Carvalho Alves Nogueira, Milton Henriques Guimarães Júnior, Patricia Klarmann Ziegelmann, Rafaela Charão de Almeida, Saionara Cristina Francisco, Sidney Teodoro Silveira Neto, Silvia Ferreira Araújo, Thiago Junqueira Avelino-Silva, Márlon Juliano Romero Aliberti, Magda Carvalho Pires, Eduardo Sérgio da Silva, Milena Soriano Marcolino

**Affiliations:** a Universidade Federal de São João del-Rei, Campus Centro Oeste Dona Lindu, Divinópolis, MG, Brazil; b Instituto de Avaliação de Tecnologia em Saúde (IATS), Porto Alegre, RS, Brazil; c Department of Internal Medicine, Medical School, Universidade Federal de Minas Gerais, Belo Horizonte, MG, Brazil; d Faculdade de Ciências Médicas de Minas Gerais, Belo Horizonte, MG, Brazil; e Hospital Eduardo de Menezes, Belo Horizonte, MG, Brazil; f Department of Statistics, Universidade Federal de Minas Gerais, Belo Horizonte, MG, Brazil; g Hospital Universitário de Santa Maria, Santa Maria, RS, Brazil; h Hospital Risoleta Tolentino Neves, Belo Horizonte, MG, Brazil; i Hospital Regional Antônio Dias, Patos de Minas, MG, Brazil; j Hospital de Clínicas de Porto Alegre, Porto Alegre, RS, Brazil; k Hospital Santo Antônio, Curvelo, MG, Brazil; l Hospital Nossa Senhora da Conceição, Porto Alegre, RS, Brazil; m Hospital Cristo Redentor, Porto Alegre, RS, Brazil; n Hospital Julia Kubitschek, Belo Horizonte, MG, Brazil; o Hospital Santa Casa de Misericórdia de Belo Horizonte, Belo Horizonte, MG, Brazil; p Hospital UNIMED BH, Belo Horizonte, MG, Brazil; q Hospital Mãe de Deus, Porto Alegre, RS, Brazil; r Hospital Universitário Canoas, Canoas, RS, Brazil; s Hospital Metropolitano Odilon Behrens, Belo Horizonte, MG, Brazil; t Hospital Bruno Born, Lajeado, RS, Brazil; u Hospital Moinhos de Vento, Porto Alegre, RS, Brazil; v Hospital Santa Cruz, Santa Cruz do Sul, RS, Brazil; w Hospital São Lucas PUCRS, Porto Alegre, RS, Brazil; x Hospitais da Rede Mater Dei, Belo Horizonte, MG, Brazil; y Hospital Márcio Cunha, Ipatinga, MG, Brazil; z Hospital Tacchini, Bento Gonçalves, RS, Brazil; aa Hospital Metropolitano Doutor Célio de Castro, Belo Horizonte, MG, Brazil; bb Medical School, Universidade Federal de São João Del-Rei, Divinópolis, MG, Brazil; cc Hospital Semper, Belo Horizonte, MG, Brazil; dd Laboratório de Investigação Médica em Envelhecimento (LIM-66), Serviço de Geriatria, Hospital das Clínicas HCFMUSP, Medical School, Universidade de São Paulo, São Paulo, SP, Brazil; ee Faculdade Israelita de Ciências da Saúde Albert Einstein, Hospital Israelita Albert Einstein, São Paulo, SP, Brazil; ff Research Institute, Hospital Sírio-Libanês, São Paulo, SP, Brazil; gg Telehealth Center, University Hospital, Universidade Federal de Minas Gerais, Belo Horizonte, MG, Brazil

**Keywords:** COVID-19, HIV, Intensive care unit, Mechanical ventilation, Mortality

## Abstract

**Objective:**

To evaluate clinical characteristics and outcomes of COVID-19 patients infected with HIV, and to compare with a paired sample without HIV infection.

**Methods:**

This is a substudy of a Brazilian multicentric cohort that comprised two periods (2020 and 2021). Data was obtained through the retrospective review of medical records. Primary outcomes were admission to the intensive care unit, invasive mechanical ventilation, and death. Patients with HIV and controls were matched for age, sex, number of comorbidities, and hospital of origin using the technique of propensity score matching (up to 4:1). They were compared using the Chi-Square or Fisher's Exact tests for categorical variables and the Wilcoxon for numerical variables.

**Results:**

Throughout the study, 17,101 COVID-19 patients were hospitalized, and 130 (0.76%) of those were infected with HIV. The median age was 54 (IQR: 43.0;64.0) years in 2020 and 53 (IQR: 46.0;63.5) years in 2021, with a predominance of females in both periods. People Living with HIV (PLHIV) and their controls showed similar prevalence for admission to the ICU and invasive mechanical ventilation requirement in the two periods, with no significant differences. In 2020, in-hospital mortality was higher in the PLHIV compared to the controls (27.9% vs. 17.7%; p = 0.049), but there was no difference in mortality between groups in 2021 (25.0% vs. 25.1%; p > 0.999).

**Conclusions:**

Our results reiterate that PLHIV were at higher risk of COVID-19 mortality in the early stages of the pandemic, however, this finding did not sustain in 2021, when the mortality rate is similar to the control group.

## Highlights

• Signs and symptoms of COVID-19 at hospital admission are similar between patients infected with HIV and controls.
• Mortality from COVID-19 in patients infected with HIV was higher compared to the controls in 2020, but no difference in 2021.
• Similar rates of ICU and invasive mechanical ventilation were observed in the different waves.

## Introduction

The Coronavirus Disease 2019 (COVID-19) pandemic has significantly impacted the assistance services offered to People Living with Human Immunodeficiency Virus (PLHIV). The introduction of restrictive measures aimed at slowing COVID-19 dissemination has resulted in reduced access to PLHIV to specialized care.^1^ This is an alarming scenario, due to the important contribution of regular follow-up towards declining the morbidity and mortality of PLHIV.^2^

Evidence demonstrates higher susceptibility to severe COVID-19 in PLHIV with uncontrolled infection due to the lower CD4+ T cells count in these patients.^3^, ^4^, ^5^, ^6^ There is evidence that the SARS-CoV-2 binds to CD4^+^ T-cells via the Angiotensin-Converting Enzyme 2 (ACE2) receptor, and it replicates in a wide range of T-helper cells. This process of viral replication induces the death of defense cells and compromises the immune system. Findings also indicate the expression of a large amount of cytokine by SARS-CoV-2 infected CD4^+^ T-cells, which is markedly associated with viral persistence and disease severity.^7^ Furthermore, it is known that pre-existing underlying diseases, commonly observed due to increased survival in PLHIV, are considered potential risk factors for the severity of COVID-19 in this population.^8^

A recent systematic review with data from over 20 million COVID-19 patients from Africa, Asia, Europe, and North America showed an increased mortality risk in PLHIV and a higher risk of hospitalization for COVID-19 among those without viral suppression and in a more advanced stage of HIV infection.^5^ Data from other meta-analyses that grouped studies developed in Africa, European, China, India, and the United States corroborate these findings.^9^^,^^10^ However, another systematic review involving seven studies found no increased risk of worse COVID-19 outcomes among PLHIV.^11^ On the other hand, some studies indicate that PLHIV are subject to lower infection rates and lower risk of developing severe forms of the disease, as a consequence of the activity attributed to the use of antiretroviral drugs.^12^, ^13^, ^14^ Divergences about the severity of COVID-19 in PLHIV remain prominent in the current scenario.

So far, no robust studies have been developed to investigate the influence of HIV infection on the outcomes of COVID-19 in Latin America in different waves of the pandemic. This continent was severely hit by the pandemic,^15^ with higher mortality when compared to countries in other regions.^16^ It is believed that the higher COVID-19 mortality in Latin America is a result of different factors, including high population density, precarious sanitary conditions, and low socioeconomic and educational levels, which are usually associated with a higher prevalence of chronic comorbidities and delays in seeking care.^16^

Given the divergences in the clinical course of COVID-19 in PLHIV and the limited knowledge in this regard in different waves, further evidence from large cohorts is still required to investigate the profile of the disease in this specific population, especially in Latin America. Therefore, the present study aimed to evaluate clinical characteristics and outcomes in patients with SARS-CoV-2 and HIV coinfection, as well as to compare their clinical outcomes to COVID-19 patients without HIV infection in different periods.

## Methods

### Study design and setting

The present study design followed the Strengthening the Report of Observational Studies in Epidemiology (STROBE) recommendations, to cover the essential items for the description of observational studies. It is a substudy of two large Brazilian cohorts. The Brazilian COVID-19 Registry is a multicenter retrospective cohort involving 37 public and private hospitals, which comprised two periods: March to September 2020 and March to December 2021. This cohort included consecutive adult patients (≥ 18 years old) with laboratory-confirmed COVID-19 admitted to the participating hospitals. The CO-FRAIL (COVID-19 and Frailty) Study was a cohort developed at the University Hospital of the University of Sao Paulo Medical School (HCFMUSP), which included consecutive patients ≥ 50 years old with laboratory-confirmed COVID-19 admitted to the hospital in March to July 2020. Details about the research setting of both registries are described in previous publications.^17^^,^^18^

Patients who manifested COVID-19 while admitted for other reasons or those transferred to other hospitals who had no outcome (discharged or death) were excluded from the present analysis.

### Data collection

In the Brazilian COVID-19 Registry, data collection was performed from the medical records by healthcare professionals properly trained to obtain the variables of interest in the study. Data were managed using the Research Electronic Data Capture (REDCap). The REDCap was hosted at the Telehealth Center, University Hospital, Universidade Federal de Minas Gerais, Belo Horizonte, Minas Gerais, Brazil.^17^ A coding manual guiding data collection was provided to the researchers involved and remained accessible for questions throughout the collection period. The outliers and missing information were verified and corrected by local references from each hospital, to ensure data reliability. At the HCFMUSP cohort, data were extracted after a detailed review of electronic medical records, nursing records, and consulting notes. These records included information documented by frontline health professionals in standardized electronic forms, specially designed for the pandemic.^18^

In both cohorts, information on sociodemographic characteristics, comorbidities, clinical assessment, laboratory findings, treatment, and outcomes was obtained. Specific variables such as CD4^+^ T-lymphocyte count, viral load, and antiretroviral therapy were also obtained for all PLHIV through the Brazilian official records: Laboratory Tests Control System (SISCEL) and Logistic Control System of Medication (SICLOM).

### Outcomes

Primary outcomes were admission to the Intensive Care Unit (ICU), invasive mechanical ventilation, and death. The secondary outcomes were other clinical complications such as renal replacement therapy requirement, adult respiratory distress syndrome, septic shock, nosocomial infection, acute myocardial infarction, deep vein thrombosis, and pulmonary thromboembolism.

### Statistical analysis

Initially, a descriptive analysis of the population was performed, in which sociodemographic and clinical characteristics of the patients under study were represented by frequency distribution, measures of central tendency, and variability. The Kolmogorov-Smirnov test was applied to verify data normality.

COVID-19 patients infected with HIV and COVID-19 patients without concomitant diagnosis of HIV infection were matched for age, sex, number of comorbidities, and hospital of admission by propensity score matching using the nearest neighbor algorithm (0.25 standard deviations of the logit of the propensity score, on a scale from 0‒1.00), up to 4:1. Groups were compared to using the Chi-Square Test and Fisher's exact test for categorical variables, and the Wilcoxon test for continuous variables. Statistical analysis was performed with R software (version 4.0.2). Results were considered statistically significant at a level of p<0.05.

### Ethics statement

The Brazilian National Commission for Research Ethics (CAAE 30350820.5.1001.0008) and the Research Ethics Committee of participating institutions approved the study protocol. Individual informed consent was waived due to the pandemic situation and the use of data from unidentified medical records. Additionally, administrative permissions to access and use the medical records were obtained from each institution. This study was performed in line with the principles of the Declaration of Helsinki.

## Results

**Fig. 1:**
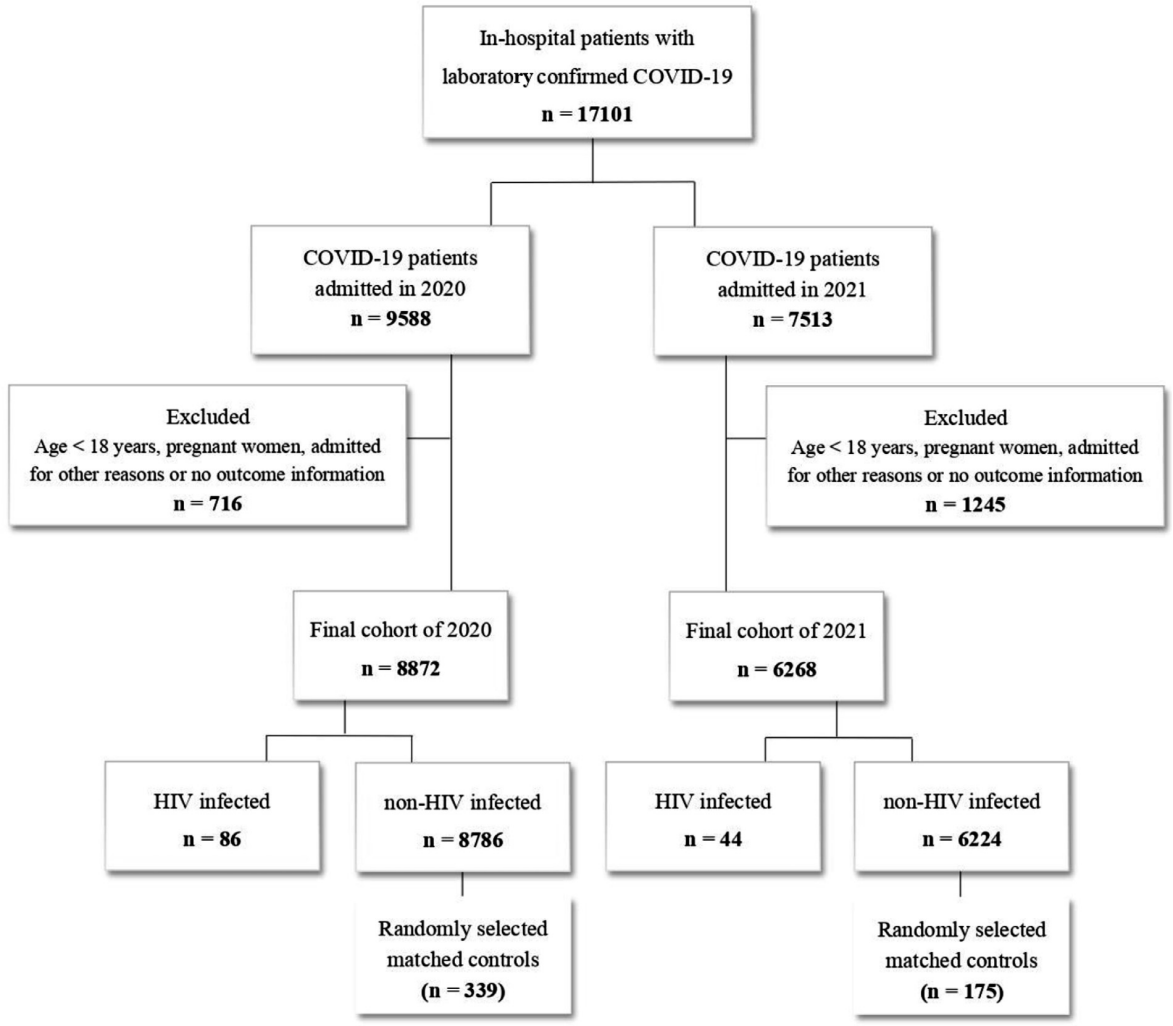
Flowchart representative of the study population.

The study included 17,101 COVID-19 patients, 130 (0.76%) were PLHIV. Of these, 86 were admitted in 2020 (0.50% of the total; median age 55 years; 62.8% women), and 44 were admitted in 2021 (0.26% of the total; median age 53 years; 59.1% women) (Fig. 1).

Table 1 shows the sociodemographic and clinical characteristics of study patients. PLHIV and matched controls had a similar frequency of comorbidities in both periods, except for a lower frequency of obesity (5.8% vs. 16.8%; p = 0.016), and a higher frequency of chronic obstructive pulmonary disease (9.3% vs. 3.8%; p = 0.049), and cancer (15.1% vs. 7.1%; p = 0.032) among those with HIV, in 2020. Regarding lifestyle habits, smoking was more frequent among PLHIV in both periods (12.8% vs. 4.7%, p = 0.013 in 2020; 13.6% vs. 4.0%, p=0.027 in 2021). Specific data related to HIV infection is shown in Supplementary Table 1.

**Table 1:** Sociodemographic characteristics and previous clinical history of patient's study.

	2020				2021			
Variables	Total (n = 425)	HIV infected (n = 86)	non-HIV infected (n = 339)	p-value	Total (n = 219)	HIV infected (n = 44)	non-HIV infected (n = 175)	p-value
	n (%)	n (%)	n (%)		n (%)	n (%)	n (%)	
**Demographic data**								
Age (years)^a^	54.0 (43.0, 64.0)	55.0 (47.0, 64.0)	54.0 (43.0, 63.5)	0.584	53.0 (46.0, 63.5)	53.0 (44.0, 59.5)	53.0 (46.0, 64.5)	0.789
Female	252 (59.3%)	54 (62.8%)	198 (58.4%)	0.538	119 (54.3%)	26 (59.1%)	93 (53.1%)	0.590
**Lifestyle habits**								
Current smoker	27 (6.4%)	11 (12.8%)	16 (4.7%)	0.013	13 (5.9%)	6 (13.6%)	7 (4.0%)	0.027
Previous smoker	61 (14.4%)	17 (19.8%)	44 (13.0%)	0.152	20 (9.1%)	5 (11.4%)	15 (8.6%)	0.562
**Comorbidities**								
Hypertension	162 (38.1%)	30 (34.9%)	132 (38.9%)	0.571	97 (44.3%)	18 (40.9%)	79 (45.1%)	0.737
Coronary artery disease	19 (4.5%)	7 (8.1%)	12 (3.5%)	0.079	7 (3.2%)	1 (2.3%)	6 (3.4%)	>0.999
Heart failure	21 (4.9%)	5 (5.8%)	16 (4.7%)	0.780	7 (3.2%)	2 (4.5%)	5 (2.9%)	0.630
Atrial fibrillation/flutter	9 (2.1%)	2 (2.3%)	7 (2.1%)	>0.999	2 (0.9%)	0 (0.0%)	2 (1.1%)	>0.999
Stroke	9 (2.1%)	3 (3.5%)	6 (1.8%)	0.395	6 (2.7%)	1 (2.3%)	5 (2.9%)	>0.999
Diabetes mellitus	88 (20.7%)	21 (24.4%)	67 (19.8%)	0.422	45 (20.5%)	8 (18.2%)	37 (21.1%)	0.821
Obesity	62 (14.6%)	5 (5.8%)	57 (16.8%)	0.016	44 (20.1%)	8 (18.2%)	36 (20.6%)	0.886
Asthma	38 (8.9%)	5 (5.8%)	33 (9.7%)	0.354	16 (7.3%)	3 (6.8%)	13 (7.4%)	>0.999
Chronic obstructive pulmonary disease	21 (4.9%)	8 (9.3%)	13 (3.8%)	0.049	7 (3.2%)	2 (4.5%)	5 (2.9%)	0.630
Cancer	37 (8.7%)	13 (15.1%)	24 (7.1%)	0.032	6 (2.7%)	3 (6.8%)	3 (1.7%)	0.097
Cirrhosis	3 (0.7)	0 (0.0%)	3 (0.9%)	>0.999	2 (0.9%)	1 (2.3%)	1 (0.6%)	0.362
Chronic kidney disease	33 (7.8%)	10 (11.6%)	23 (6.8%)	0.203	5 (2.3%)	1 (2.3%)	4 (2.3%)	>0.999
Rheumatological disease	7 (1.6%)	1 (1.2%)	6 (1.8%)	>0.999	4 (1.8%)	1 (2.3%)	3 (1.7%)	>0.999
Psychiatric illness	21 (4.9%)	9 (10.5%)	12 (3.5%)	0.021	17 (7.8%)	5 (11.4%)	12 (6.9%)	0.345
**Number of comorbidities**								
0	171 (40.2%)	34 (39.5%)	137 (40.4%)		78 (35.6%)	17 (38.6%)	61 (34.9%)	
1	132 (31.1%)	24 (27.9%)	108 (31.9%)		80 (36.5%)	15 (34.1%)	65 (37.1%)	
2	85 (20.0%)	19 (22.1%)	66 (19.5%)	0.810	43 (19.6%)	8 (18.2%)	35 (20.0%)	0.910
3	27 (6.4%)	6 (7.0%)	21 (6.2%)		15 (6.8%)	3 (6.8%)	12 (6.9%)	
4	10 (2.3%)	3 (0.7%)	7 (1.6%)		3 (1.4%)	1 (2.3%)	2 (1.1%)	

a Median (Interquartile Range ‒ IQR). Statistical tests: Wilcoxon rank-sum test; Chi-Square test of independence; Fisher's exact test.

Dyspnea (65.6% in 2020; 69.4% in 2021) and fever (60.5% in 2020; 43.8% in 2021) were the most frequent symptoms in both groups and both periods, with no significant differences (Supplementary Table 2). Concerning the laboratory exams, HIV-infected patients admitted in 2020 had lower counts of leukocytes (5,950.0 [4,595.0, 8,220.0] vs. 7,370.5 [5,415.0, 10,257.5]; p < 0.001) and neutrophils (4,303.5 [2,715.0, 6,088.0] vs 5,435.5 [3,658.8, 8,015.0]; p = 0.001), in which HIV infected patients had lower counts compared to controls. Alanine aminotransferase level (30.0 [22.7, 42.0] vs. 37.0 [23.0, 63.8]; p = 0.021) was also lower among HIV-infected patients. These differences were observed among patients admitted in 2021 (Table 2).

**Table 2:** Clinical and laboratory assessment at hospital admission.

	2020				2021			
Variables	Total (n = 425)	HIV infected (n = 86)	non-HIV infected (n = 339)	p-value	Total (n = 219)	HIV infected (n = 44)	non-HIV infected (n = 175)	p-value
	n (%)	n (%)	n (%)		n (%)	n (%)	n (%)	
Clinical assessment^a^								
Glasgow <15	79 (18.6%)	16 (18.6%)	63 (18.6%)	>0.999	11 (5.0%)	3 (6.8%)	8 (4.6%)	0.465
Heart rate (bpm)	90.0 (79.0, 101.2)	88.5 (77.8, 101.2)	90.0 (79.8, 101.2)	0.410	86.0 (77.8, 95.2)	86.0 (80.0, 95.0)	86.0 (76.0, 95.0)	0.666
Respiratory rate (irpm)	20.0 (18.0, 25.0)	20.0 (19.0, 24.0)	20.5 (18.0, 25.2)	0.859	20.0 (18.0, 24.0)	22.0 (18.0, 26.0)	20.0 (18.8, 24.0)	0.608
SF ratio	411.0 (247.9, 456.0)	431.0 (254.6, 458.3)	407.5 (247.9, 452.4)	0.316	342.9 (275.3, 419.0)	344.6 (276.7, 422.6)	339.3 (275.3, 419.0)	0.741
Mechanical ventilation	54 (12.7%)	11 (12.8%)	43 (12.7%)	>0.999	2 (1.1%)	2 (5.6%)	0 (0.0%)	0.041
Systolic blood pressure								
≥90 (mm Hg)	369 (91.8%)	73 (91.2%)	296 (91.9%)		166 (97.1%)	33 (97.1%)	133 (97.1%)	
<90 (mm Hg)	5 (1.2%)	1 (1.2%)	4 (1.2%)	0.921	2 (1.2%)	0 (0.0%)	2 (1.5%)	0.675
Inotrope requirement	28 (7.0%)	6 (7.5%)	22 (6.8%)		3 (1.8%)	1 (2.9%)	2 (1.5%)	
Diastolic blood pressure								
>60 (mm Hg)	319 (79.4%)	62 (77.5%)	257 (79.8%)		143 (83.6%)	27 (79.4%)	116 (84.7%)	
≤60 (mm Hg)	55 (13.7%)	12 (15.0%)	43 (13.4%)	0.900	25 (14.6%)	6 (17.6%)	19 (13.9%)	0.542
Inotrope requirement	28 (7.0%)	6 (7.5%)	22 (6.8%)		3 (1.8%)	1 (2.9%)	2 (1.5%)	
**Laboratory assessment ^1^**								
*Hemogram parameters*								
Hemoglobin (g/dL)	13.1 (11.4, 14.4)	12.7 (10.6, 14.2)	13.2 (11.6, 14.4)	0.149	13.4 (12.3, 14.6)	13.4 (12.1, 14.8)	13.3 (12.4, 14.5)	0.919
Leukocytes (cels/mm^3^)	7,100.0 (5,100.0, 9,850.0)	5,950.0 (4,595.0, 8,220.0)	7,370.5 (5,415.0, 10,257.5)	<0.001	6,890.0 (5,765.0, 9,310.0)	6,770.0 (5,840.0, 8,430.0)	6,941.0 (5,597.0, 9,310.0)	0.551
Neutrophiles (cels/mm^3^)	5,219.0 (3,420.5, 7,666.5)	4,303.5 (2,715.0, 6,088.0)	5,435.5 (3,658.8, 8,015.0)	0.001	5,301.2 (3,966.8, 7,331.2)	4,989.0 (4,194.4, 6,718.0)	5,410.0 (3,849.8, 7,392.0)	0.692
Lymphocytes (cels/mm^3^)	1,000.0 (710.0, 1,382.0)	1,013.0 (593.2, 1,398.2)	1,000.0 (737.0, 1,380.0)	0.500	978.5 (673.8, 1,492.5)	965.0 (508.0, 1,434.0)	994.0 (684.0, 1,499.0)	0.374
Platelet count (10^9^/L)	199,000.0 (158,000.0, 266,750.0)	182,000.0 (147,500.0, 251,500.0)	205,000.0 (162,000.0, 268,000.0)	0.089	214,500.0 (159,500.0, 273,250.0)	219,000.0 (178,500.0, 262,500.0)	210,000.0 (158,000.0, 274,000.0)	0.700
NLR (cels/mm^3^)	5.1 (3.0, 8.7)	4.4 (2.5, 7.5)	5.3 (3.0, 8.9)	0.142	5.6 (3.5, 9.1)	6.2 (3.9, 10.6)	5.3 (3.4, 8.8)	0.466
*Kidney parameters*								
Creatinine (mg/dL)	0.9 (0.7, 1.2)	0.9 (0.7, s1.4)	0.9 (0.7, 1.2)	0.530	0.8 (0.7, 1.1)	0.9 (0.7, 1.2)	0.8 (0.7, 1.0)	0.340
Urea (mg/dL)	35.0 (25.0, 52.3)	35.5 (24.0, 55.6)	35.0 (26.0, 50.8)	0.856	32.6 (26.0, 45.8)	37.0 (26.2, 54.7)	31.7 (26.0, 44.2)	0.249
*Liver parameters*								
AST (U/L)	41.0 (30.0, 61.0)	37.0 (27.8, 53.0)	42.0 (30.0, 63.0)	0.090	47.2 (36.2, 69.7)	41.0 (32.5, 58.7)	51.8 (39.2, 69.8)	0.066
ALT (U/L)	34.0 (23.0, 58.0)	30.0 (22.7, 42.0)	37.0 (23.0, 63.8)	0.021	44.0 (28.0, 72.0)	42.0 (24.0, 54.5)	47.5 (32.0, 72.8)	0.139
*Ions*								
Sodium (mmoL/L)	138.0 (135.0, 141.0)	138.0 (135.0, 140.0)	138.0 (136.0, 141.0)	0.291	137.0 (134.0, 139.0)	137.0 (134.8, 139.0)	137.0 (134.0, 139.0)	0.868
*Others parameters*								
INR	1.1 (1.0, 1.2)	1.1 (1.0, 1.2)	1.1 (1.0, 1.2)	0.652	1.1 (1.0, 1.2)	1.1 (1.0, 1.2)	1.1 (1.0, 1.1)	0.181
Lactate (mmoL/L)	1.3 (0.9, 1.8)	1.5 (1.1, 1.9)	1.2 (0.9, 1.6)	0.006	1.5 (1.1, 2.2)	1.8 (1.3, 2.3)	1.5 (1.1, 2.1)	0.219
Arterial pH	7.4 (7.4, 7.5)	7.4 (7.4, 7.4)	7.4 (7.4, 7.5)	0.013	7.4 (7.4, 7.5)	7.4 (7.4, 7.5)	7.4 (7.4, 7.5)	0.759
Arterial pO_2_	77.0 (63.2, 96.0)	79.0 (60.8, 104.8)	76.1 (63.8, 94.1)	0.381	71.4 (61.5, 95.0)	77.0 (65.1, 99.7)	70.5 (61.0, 95.0)	0.192
Arterial pCO_2_	35.5 (32.0, 40.0)	35.2 (30.0, 42.0)	35.8 (32.1, 39.2)	0.659	35.0 (31.6, 38.2)	33.6 (30.0, 35.6)	35.9 (32.0, 39.0)	0.046
Bicarbonate	23.6 (21.0, 25.9)	23.0 (19.0, 25.2)	23.8 (21.3, 26.0)	0.073	23.2 (21.1, 25.1)	22.0 (19.6, 23.8)	24.0 (22.0, 25.3)	0.002

a Median (Interquartile Range ‒ IQR). Statistical tests: Wilcoxon rank-sum test; Chi-Square test of independence; Fisher's exact test. INR, International Normalized Ratio; NLR, Neutrophil to Lymphocyte Ratio; SF, Ratio – Peripheral; SpO_2_/FiO_2_ ratio, Capillary Oxygen Saturation/Fraction of Inspired Oxygen.

In both periods, no significant differences were found between HIV-infected patients and controls for any of the medications or supportive care during the hospital stay (Supplementary Table 3). There was higher mortality among patients with HIV in 2020 when compared to matched controls (27.9% vs. 60 17.7%; p = 0.049), with no difference in other clinical outcomes. In 2021, there was no difference in outcomes between groups (Table 3).

**Table 3:** Clinical outcomes during hospitalization.

	2020				2021			
Variables	Total (n = 425)	HIV infected (n = 86)	non-HIV infected (n = 339)	p-value	Total (n = 219)	HIV infected (n = 44)	non-HIV infected (n = 175)	p-value
	n (%)	n (%)	n (%)		n (%)	n (%)	n (%)	
**Outcomes**								
Septic shock	81 (19.1%)	18 (20.9%)	63 (18.6%)	0.733	31 (14.2%)	6 (13.6%)	25 (14.3%)	>0.999
Adult respiratory distress syndrome	88 (20.7%)	16 (18.6%)	72 (21.2%)	0.697	71 (32.4%)	15 (34.1%)	56 (32.0%)	0.932
Acute myocardial infarction	6 (1.4%)	2 (2.3%)	4 (1.2%)	0.350	2 (0.9%)	0 (0.0%)	2 (1.1%)	>0.999
Nosocomial infection	73 (17.2%)	14 (16.3%)	59 (17.4%)	0.931	41 (18.7%)	9 (20.5%)	32 (18.3%)	0.910
Deep vein thrombosis	12 (2.8%)	2 (2.3%)	10 (2.9%)	>0.999	6 (2.7%)	1 (2.3%)	5 (2.9%)	>0.999
Pulmonary thromboembolism	24 (5.6%)	4 (4.7%)	20 (5.9%)	0.798	15 (6.8%)	6 (13.6%)	9 (5.1%)	0.086
Renal replacement therapy requirement	53 (12.5%)	13 (15.1%)	40 (11.8%)	0.516	26 (11.9%)	6 (13.6%)	20 (11.4%)	0.885
Admission to the ICU	178 (42.0%)	37 (43.5%)	141 (41.6%)	0.841	94 (43.1%)	17 (38.6%)	77 (44.3%)	0.616
Length of stay (days)^a^	9.0 (4.0, 21.0)	9.0 (4.0, 18.0)	9.0 (4.0, 23.0)	0.512	10.5 (5.0, 16.8)	11.0 (5.0, 18.0)	10.0 (5.0, 16.0)	0.906
Mechanical ventilation support	136 (32.5%)	30 (35.7%)	106 (31.7%)	0.572	71 (32.4%)	15 (34.1%)	56 (32.0%)	0.932
Death	84 (19.8%)	24 (27.9%)	60 (17.7%)	0.049	55 (25.1%)	11 (25.0%)	44 (25.1%)	>0.999

a Median (Interquartile Range ‒ IQR). Statistical tests: Wilcoxon rank-sum test; Chi-Square test of independence; Fisher's exact test.

## Discussion

This study presents the clinical characteristics and outcomes of COVID-19 in PLHIV and matched controls, admitted in participating hospitals from 14 Brazilian cities in 2020 and 2021. Clinical characteristics were similar between groups, except for comorbidities such as obesity, chronic obstructive pulmonary disease, and cancer and laboratory results of total leukocytes and neutrophils, which showed differences in the first period under analysis. Although patients with HIV admitted in 2020 had higher mortality than matched controls, this finding has not been observed among those admitted in 2021.

Despite the conflicting evidence regarding the influence of HIV infection on the clinical course and outcome of COVID-19, most studies – including systematic reviews and meta-analyses – agree that PLHIV has a higher risk of death from COVID-19.^5^^,^^9^^,^^10^^,^^19^, ^20^, ^21^, ^22^ However, a thorough analysis of the literature demonstrates that most of the available studies comprise samples from 2020 when there were a large number of publications due to the urgency of updates on COVID-19. These studies have not included the vaccination period, as the World Health Organization issued the first document supporting the approval of a COVID-19 vaccine on December 31, 2020. Additionally, the most recent studies evaluating COVID-19 outcomes in PLHIV include data collected no later than July 2021 and therefore do not cover a more advanced stage of COVID-19 vaccination.^6^^,^^19^^,^^20^^,^^22^, ^23^, ^24^

Although the vaccination program in Brazil began in January 2021, the immunization rates advanced slowly in the first half of that year due to the limited availability of vaccines. By the end of 2021, with a significant advance in immunization, the country had reached the coverage of 67.9% of the population fully vaccinated and 78.2% with at least one vaccine dose.^25^ Regarding the definition of target groups for immunization by the Ministry of Health, the PLHIV were considered a priority if they had a CD4^+^ count < 350 mm^3^, otherwise, the vaccination followed the age criteria, with older people being vaccinated first.^26^ In our cohort, which includes patients admitted up to December 2021, the mortality rate in PLHIV was stable in both periods (before and after the vaccination program started in Brazil).

Regarding specific information related to HIV infection, the authors obtained data from 99 (76.1%) patients in the study. Most patients with available data were on antiretroviral therapy (100.0%) and had suppressed viral loads (2020: 78.1% and 2021: 71.4%). Similarly, in a meta-analysis that analyzed the outcomes of COVID-19 in HIV-infected individuals from seven countries, 96.0% were on antiretroviral therapy, and about 80.0% were suppressed.^5^ Brazil is a country that stands out in Latin America for a strong HIV response, with an established national public program designated for the identification, monitoring, surveillance, and access to free antiretrovirals since the 1990s.^27^ Currently, 650,000 PLHIV are on antiretroviral therapy and 66.0% have suppressed viral load in the country, observed constant efforts to reach the global goal of viral suppression to 90.0%.^28^

The present results also showed that only 54.7% of PLHIV admitted in 2020, with available data, had CD4^+^ T-lymphocyte counts above 500 cells/µL, a condition that may have contributed to the finding of greater severity of COVID-19 among PLHIV compared to controls. Notably, the immune dysregulation resulting from HIV infection is a contributing factor to the severity of COVID-19.^6^ According to Davanzo et al.,^7^ the binding of SARS-CoV-2 to the defense cells of patients with HIV coinfection is capable of inducing a pronounced cell death process. Liu et al.^29^ and Hoffman et al.^4^ also have described an exacerbated expression of cytokines and chemokines in patients with SARS-CoV-2 and HIV coinfection, which might trigger a hyperinflammatory response and potentiate the negative outcomes of COVID-19. Furthermore, it is known that immunocompromise may be established before the initiation of antiretroviral therapy and, therefore, even with treatment adherence, HIV-infected patients may find themselves in a state of persistent immune dysregulation and, consequently, present a higher risk of severity during the COVID-19 course.^3^

In 2021, 70.0% of PLHIV, with available data, had a CD4^+^ T-lymphocyte count greater than or equal to 500 cells/μL. The clinical trial developed by Frater et al.,^30^ provides clear evidence of the efficacy and immunogenicity of vaccination against COVID-19 for PLHIV with CD4^+^ T-lymphocyte counts greater than 350 cells/μL. Despite high CD4^+^ T-lymphocyte counts in 2021, the study design does not allow concluding that vaccination is responsible for the absence of difference in mortality between patients living with HIV and the control group in 2021.

Information regarding the clinical history obtained for patients admitted for COVID-19 in 2020 indicates that PLHIV had a higher frequency of comorbidities, such as chronic obstructive pulmonary disease (9.3% vs. 3.8%; p = 0.049) and cancer (15.1% vs. 7.1%; p = 0.032) than patients not infected with HIV. According to Maciel et al.,^31^ PLHIV are susceptible to premature aging as a result of the inflammatory process associated with HIV infection and, even at younger ages, have a higher prevalence of chronic conditions common in older populations not infected with the virus. Therefore, it is believed that advanced age and the presence of a greater number of comorbidities are capable of influencing the severity of COVID-19 in PLHIV, which may explain the higher mortality observed in this specific population in 2020.^5^^,^^32^ According to this standard, the authors observe that in 2021, both the comorbidities and mortality rates were similar in both groups.

During the assessment of aspects related to lifestyle habits, PLHIV showed a higher frequency of smoking compared to controls in both periods (12.8% vs. 4.7%, p = 0.013 in 2020; 13.6% vs. 4.0%, p = 0.027 in 2021). There is concrete evidence that PLHIV is two to three times more exposed to smoking than the general population.^33^ Studies involving patients with COVID-19 demonstrate greater disease severity among smokers.^34^ Thus, the higher frequency of comorbidities such as chronic obstructive pulmonary disease and cancer, as well as the high number of smokers among PLHIV in this study, may have an influence on findings of higher mortality in this population compared to patients not infected with HIV.

As for clinical signs and symptoms upon hospital presentation, the findings were similar between both groups, with dyspnea and fever being the most prevalent symptoms. These findings are in line with previous studies and reinforce that the screening and clinical suspicion of COVID-19 for the HIV-infected population should be similar to the general population.^35^ Clinical assessment characteristics upon hospital presentation were similar for HIV-infected and non-HIV-infected patients in both study periods. Laboratory findings also were mostly similar to non-HIV-infected patients. Despite the lower leukocytes and neutrophil counts compared to the non-infected population, that difference did not maintain in 2021. Although chronic HIV infection might lead to lower neutrophil counts,^36^ it is unclear the clinical significance of this finding in the context of an acute SARS-CoV-2 infection.

Most patients included in both study periods were on antiretroviral therapy, with lamivudine (96.9%), tenofovir (61.5%) and dolutegravir (57.3%) being the most used antiretrovirals. There is evidence of the benefit of antiretrovirals such as tenofovir against COVID-19.^13^^,^^14^ In vitro and molecular docking studies suggest that tenofovir inhibits the Ribonucleic Acid (RNA)-dependent RNA polymerase of SARS-CoV-2.^37^ This antiretroviral also has immunomodulatory effects, including decreased production of Interleukin (IL)-8 and IL-10,^38^ cytokines that are associated with COVID-19 severity and mortality.^29^

Concerning the study limitations, the sample of PLHIV was small, which indicates the need for a careful interpretation of the results. Even so, the point estimate for mortality in 2021 was approximately the same for patients with HIV and matched controls. Despite the restricted sample size, this study presents great relevance since to the best of our knowledge there are no publications directed to the investigation of patients with coinfection by SARS-COV-2 and HIV in Latin America in different waves of the pandemic. In this context, it is noticeable that knowledge about the clinical course of COVID-19 in this specific population is crucial to ensure the appropriate management of the disease, as well as to improve the management of health care costs, which is relevant for many countries of the continent that had great restriction of resources during the COVID-19 pandemic.^39^ Another limitation is due to the retrospective study design. Information such as the vaccination status of patients and specific data related to HIV infection could not be fully recovered in the hospital records and other records under analysis. The resources used to obtain the variables of interest in the study are secondary sources of information, and since they were not specifically designed for data collection, the absence of indispensable information for the study is inevitable. Additionally, the work overload of health professionals can make it difficult to assiduously record information about the assistance provided to the patient, directly affecting the quality of the data source.

The major advantage of this study focuses on the involvement of large Brazilian cohorts, including patients from 27 hospitals in 14 cities and 4 different states (Minas Gerais, São Paulo, Rio Grande do Sul, and Pernambuco), ensuring the diversity of the population studied. Another relevant factor is the comparison of clinical characteristics and outcomes in patients with SARS-CoV-2 and HIV co-infection in two consecutive years (2020 and 2021), which has not been observed in other cohorts published until the moment.

## Conclusions

The present results reiterate that PLHIV were at higher risk of COVID-19 mortality in the early stages of the pandemic, indicating the importance of prioritizing the clinical management of the disease in this specific population. However, this finding did not sustain in 2021, when the mortality rate is similar to the control group. The study has limitations and, therefore, further investigations are needed to elucidate the impact of HIV infection on COVID-19.

## Authors’ contributions

MSM, TLSS, and MCP had substantial contributions to the conception or design of the work. TLSS, MVRSS, PDP, JVBN, MFS, VCMA, HD, NRO, NCSS, LEFR, AVS, AOJ, ALBAS, BMC, CTCAS, CMR, FA, FAB, GMSG, GFN, KBR, LSMM, LCC, LAN, MC, MFG, MCAN, MHGJ, PKZ, RCA, SCF, STSN, SFA, TJAS, MJRA, MCP, ESS, and MSM had substantial contributions to the acquisition, analysis, or interpretation of data for the work. All authors read and approved the final manuscript.

## Declaration of Competing Interest

The authors declare no conflicts of interest.

## Appendix Supplementary materials

Image, application 1
