# Supplementary material for: COVID-19 outcomes in people living with HIV: Peering through the waves
Source: Clinics (Sao Paulo). 2023 May 25;78:100223. doi: 10.1016/j.clinsp.2023.100223 (PMC10209448; doi:10.1016/j.clinsp.2023.100223)
Supplement: Supplementary file 1 [file mmc1.docx]

**CLINICS-D-22-00691_Supplementary Material**

**Supplementary Table 1** Specific data related to HIV infection.

| **Variables** | **Total**  **(n = 99)** | **Non missing cases** | **2020**  **(n = 77)** | **Non missing cases** | **2021**  **(n = 22)** | **Non missing cases** |
| --- | --- | --- | --- | --- | --- | --- |
|  | **n (%)** |  | **n (%)** |  | **n (%)** |  |
| **Clinical characteristics** |  |  |  |  |  |  |
| Viral load (copies/mL)^a^ | 1852.5 (98.7, 445915.5) | 20 | 151.0 (66.0, 255590,5) | 14 | 20868.0 (3386.2, 1467696.5) | 6 |
| Viral load <40 copies/mL | 65 (76.5%) | 85 | 50 (78.1%) | 64 | 15 (71.4%) | 21 |
| CD4^+^ T-lymphocyte (cells/μL)^b^ | 595.7 (± 367.7) | 84 | 545.7 (± 344.7) | 64 | 755.7 (± 401.7) | 20 |
| CD4+ T-lymphocytes ≥500 cells/μL | 49 (58.3%) | 84 | 35 (54.7%) | 64 | 14 (70.0%) | 20 |
| **Pharmacoterapeutical characteristics** |  |  |  |  |  |  |
| Antiretroviral therapy | 96 (100.0%) | 96 | 74 (100.0%) | 74 | 22 (100.0%) | 22 |
| Abacavir | 15 (15.6%) | 96 | 13 (17.6%) | 74 | 2 (9.1%) | 22 |
| Atazanavir | 16 (16.7%) | 96 | 10 (13.5%) | 74 | 6 (27.3%) | 22 |
| Darunavir | 11 (11.5%) | 96 | 11 (14.9%) | 74 | 0.0% | 22 |
| Dolutegravir | 55 (57.3%) | 96 | 42 (56.8%) | 74 | 13 (59.1%) | 22 |
| Efavirenz | 17 (17.7%) | 96 | 15 (20.3%) | 74 | 2 (9.1%) | 22 |
| Etravirina | 1 (1.0%) | 96 | 1 (1.4%) | 74 | 0 (0.0%) | 22 |
| Lamivudina | 93 (96.9%) | 96 | 71 (95.9%) | 74 | 22 (100.0%) | 22 |
| Maraviroque | 1 (1.0%) | 96 | 1 (1.4%) | 74 | 0 (0.0%) | 22 |
| Nevirapina | 3 (3.1%) | 96 | 2 (2.7%) | 74 | 1 (4.5%) | 22 |
| Raltegravir | 1 (1.0%) | 96 | 1 (1.4%) | 74 | 0 (0.0%) | 22 |
| Ritonavir | 27 (28.1%) | 96 | 21 (28.4%) | 74 | 6 (27.3%) | 22 |
| Tenofovir | 59 (61.5%) | 96 | 46 (62.2%) | 74 | 13 (59.1%) | 22 |
| Zidovudina | 8 (8.3%) | 96 | 5 (6.8%) | 74 | 3 (13.6%) | 22 |
| Total number of antiretrovirals | 3 (3, 4)^a^ | 96 | 3 (3, 4)^a^ | 74 | 3 (3, 4)^a^ | 22 |
| Duration of current antiretroviral therapy (months)^a^ | 27 (10, 43) | 96 | 26 (10, 41) | 74 | 31 (5, 73) | 22 |

^a^ Median (Interquartile Range ‒ IQR). ^b^ Mean (Standard Deviation).

**Supplementary Table 2** Prevalence of symptoms at hospital admission.

|  | **2020** | | | | **2021** | | | |
| --- | --- | --- | --- | --- | --- | --- | --- | --- |
| **Variables** | **Total**  **(n = 425)** | **HIV infected**  **(n = 86)** | **non-HIV infected**  **(n = 339)** | **p-value** | **Total**  **(n = 219)** | **HIV infected**  **(n = 44)** | **non-HIV infected**  **(n = 175)** | **p-value** |
|  | **n (%)** | **n (%)** | **n (%)** |  | **n (%)** | **n (%)** | **n (%)** |  |
| **Symptoms** |  |  |  |  |  |  |  |  |
| Duration of symptoms (days)^a^ | 7.0 (3.0, 9.0) | 6.0 (3.0, 9.0) | 7.0 (4.0, 9.0) | 0.444 | 9.0 (6.0, 12.0) | 9.0 (6.0, 11.0) | 9.0 (6.0, 12.0) | 0.780 |
| Adynamia | 108 (25.4%) | 22 (25.6%) | 86 (25.4%) | >0.999 | 64 (29.2%) | 11 (25.0%) | 53 (30.3%) | 0.614 |
| Ageusia | 32 (7.5%) | 6 (7.0%) | 26 (7.7%) | >0.999 | 19 (8.7%) | 1 (2.3%) | 18 (10.3%) | 0.133 |
| Anosmia | 39 (9.2%) | 6 (7.0%) | 33 (9.7%) | 0.561 | 22 (10.0%) | 2 (4.5%) | 20 (11.4%) | 0.262 |
| Diarrhea | 61 (14.4%) | 11 (12.8%) | 50 (14.7%) | 0.771 | 37 (16.9%) | 8 (18.2%) | 29 (16.6%) | 0.976 |
| Dyspnea | 279 (65.6%) | 52 (60.5%) | 227 (67.0%) | 0.314 | 152 (69.4%) | 30 (68.2%) | 122 (69.7%) | 0.989 |
| Fever | 257 (60.5%) | 50 (58.1%) | 207 (61.1%) | 0.710 | 96 (43.8%) | 20 (45.5%) | 76 (43.4%) | 0.942 |
| Headache | 95 (22.4%) | 20 (23.3%) | 75 (22.1%) | 0.936 | 52 (23.7%) | 5 (11.4%) | 47 (26.9%) | 0.050 |
| Myalgia | 109 (25.6%) | 18 (20.9%) | 91 (26.8%) | 0.325 | 74 (33.8%) | 16 (36.4%) | 58 (33.1%) | 0.822 |
| Rhinorrhea | 37 (8.7%) | 6 (7.0%) | 31 (9.1%) | 0.672 | 31 (14.2%) | 6 (13.6%) | 25 (14.3%) | >0.999 |

^a^ Median (Interquartile Range ‒IQR). Statistical tests: Wilcoxon rank-sum test; Chi-square test of independence; Fisher's exact test.

**Supplementary Table 3** Therapeutic interventions at hospitalization.

|  | **2020** | | | | **2021** | | | |
| --- | --- | --- | --- | --- | --- | --- | --- | --- |
| **Variables** | **Total**  **(n = 425)** | **HIV infected**  **(n = 86)** | **non-HIV infected**  **(n = 339)** | **p-value** | **Total**  **(n = 219)** | **HIV infected**  **(n = 44)** | **non-HIV infected**  **(n = 175)** | **p-value** |
|  | **n (%)** | **n (%)** | **n (%)** |  | **n (%)** | **n (%)** | **n (%)** |  |
| **Medications** |  |  |  |  |  |  |  |  |
| Antibiotic to COVID-19 | 309 (72.7%) | 55 (64.0%) | 254 (74.9%) | 0.057 | 135 (61.9%) | 24 (54.5%) | 111 (63.8%) | 0.340 |
| Antibiotic to nosocomial infection | 375 (88.4%) | 77 (89.5) | 298 (88.2%) | 0.868 | 77 (35.5%) | 16 (37.2%) | 61 (35.1%) | 0.931 |
| Anticoagulant | 318 (75.0%) | 63 (73.3%) | 255 (75.4%) | 0.780 | 207 (95.4%) | 41 (95.3%) | 166 (95.4%) | >0.999 |
| Corticotherapy | 275 (64.9%) | 49 (57.0%) | 226 (66.9%) | 0.112 | 204 (94.0%) | 40 (93.0%) | 164 (94.3%) | 0.725 |
| Immunoglobulin | 1 (0.2%) | 0 (0.0%) | 1 (0.3%) | >0.999 | 0 (0.0%) | 0 (0.0%) | 0 (0.0%) | ‒ |
| **Supportive care** |  |  |  |  |  |  |  |  |
| Inotropes | 110 (25.9%) | 28 (32.6%) | 82 (24.2%) | 0.149 | 55 (25.2%) | 12 (27.3%) | 43 (24.7%) | 0.877 |
| Noninvasive mechanical ventilation | 64 (15.1%) | 14 (16.3%) | 50 (14.7%) | 0.853 | 56 (25.7%) | 10 (22.7%) | 46 (26.4%) | 0.757 |

Statistical tests: Chi-Square test of independence; Fisher's exact test.
